# Thermoplastic PCL-*b*-PEG-*b*-PCL and HDI Polyurethanes for Extrusion-Based 3D-Printing of Tough Hydrogels

**DOI:** 10.3390/bioengineering5040099

**Published:** 2018-11-14

**Authors:** Aysun Güney, Christina Gardiner, Andrew McCormack, Jos Malda, Dirk W. Grijpma

**Affiliations:** 1 Department of Biomaterials Science and Technology, Science and Technology Faculty, Technical Medical Centre, University of Twente, 7500AE Enschede, The Netherlands; a.guney@utwente.nl (A.G.); cgardine@students.uni-mainz.de (C.G.); mccormacka95@gmail.com (A.M.); 2 Department of Orthopaedics, University Medical Center Utrecht, 3584 CX Utrecht, The Netherlands; j.malda@umcutrecht.nl; 3 Regenerative Medicine Utrecht, Utrecht University, 3584 CX Utrecht, The Netherlands; 4 Faculty of Veterinary Sciences, Utrecht University, 3584 CL Utrecht, The Netherlands

**Keywords:** thermoplastic polyurethanes, tough hydrogels, 3D-printing, fused deposition modeling

## Abstract

Novel tough hydrogel materials are required for 3D-printing applications. Here, a series of thermoplastic polyurethanes (TPUs) based on poly(ɛ-caprolactone)-*b*-poly(ethylene glycol)-*b*-poly(ɛ-caprolactone) (PCL-*b*-PEG-*b*-PCL) triblock copolymers and hexamethylene diisocyanate (HDI) were developed with PEG contents varying between 30 and 70 mol%. These showed excellent mechanical properties not only when dry, but also when hydrated: TPUs prepared from PCL-*b*-PEG-*b*-PCL with PEG of Mn 6 kg/mol (PCL_7_-PEG_6_-PCL_7_) took up 122 wt.% upon hydration and had an E-modulus of 52 ± 10 MPa, a tensile strength of 17 ± 2 MPa, and a strain at break of 1553 ± 155% in the hydrated state. They had a fracture energy of 17976 ± 3011 N/mm^2^ and a high tearing energy of 72 kJ/m^2^. TPUs prepared using PEG with Mn of 10 kg/mol (PCL_5_-PEG_10_-PCL_5_) took up 534% water and were more flexible. When wet, they had an E-modulus of 7 ± 2 MPa, a tensile strength of 4 ± 1 MPa, and a strain at break of 147 ± 41%. These hydrogels had a fracture energy of 513 ± 267 N/mm^2^ and a tearing energy of 16 kJ/m^2^. The latter TPU was first extruded into filaments and then processed into designed porous hydrogel structures by 3D-printing. These hydrogels can be used in 3D printing of tissue engineering scaffolds with high fracture toughness.

## 1. Introduction

Hydrogels are water-swollen polymer networks that can be used in a variety of applications in the biomedical field, such as in biomedical devices, biomedicine, sensors, and robots [1,2]. There is a great need to develop tough biodegradable hydrogels for application in the repair and regeneration of load-bearing soft tissues like cartilage, blood vessels and tendons. These tissues take up large amounts of water, are strong and resilient, have low elasticity modulus values, and at the same time are resistant to tearing [3].

While natural hydrogel materials support cell growth, cell migration, and capillary network formation in the absence of added growth factors [3], a major limitation in their application is their lack of mechanical resilience. It remains a challenge to develop robust (natural as well as synthetic) hydrogels with suitable mechanical properties in the hydrated state [4,5].

Several approaches have been investigated to prepare tough hydrogels. These include the preparation of hydrophilic polymer networks based on co-macromer networks, double network gels, interpenetrating multi-network gels, nanocomposite gels, slip-link gels, ionic-covalent entanglement gels, etc. [6,7,8,9,10]. Tough natural hydrogel networks have been prepared from mixtures of hyaluronic acid-tyramine and chondroitin sulfate-tyramine conjugates [11].

Hydrogels play an important role in bioprinting as well, but progress in this upcoming research field is limited by the lack of suitable printable hydrogels. Difficulties in the fabrication of structures by (extrusion-based) 3D printing are often related to the brittleness of the hydrogels used, resulting in structures with poor mechanical characteristics which can only be built at low resolutions [12,13]. To our knowledge, there are no other published examples of tough hydrogel structures prepared by 3D printing.

Thermoplastic polyurethanes (TPUs) are well-known in medical applications and are processable by extrusion-based 3D printing methods. They are a versatile class of polymers with high toughness and excellent control over their properties by variation of their chemical composition [14]. Poly(ethylene glycol) (PEG) is a hydrophilic polymer that is often used in biomedical engineering, and TPUs based on PEG can be designed to form physical hydrogel networks that take up large amounts of water [15]. Such hydrogels are not prepared using potentially toxic crosslinking agents [16,17,18], and are expected to be highly compatible with cells and tissues.

In this paper, we describe the development of tough thermoplastic hydrogels based on the formation of polyurethanes by reaction of poly(ɛ-caprolactone)-*b*-poly(ethylene glycol)-*b*-poly(ɛ-caprolactone) triblock copolymers (PCL-*b*-PEG-*b*-PCL) and hexamethylene diisocyanate. In the TPU multi-block copolymer the PEG component will render the material hydrophilic, while, the hydrophobic PCL component will not only allow fixation of the structure during printing by crystallization upon cooling, but will also form the physical crosslinks of the network in the hydrated state. The literature on polyurethanes prepared from PCL-PEG-PCL triblock copolymers is very limited [19,20]. However, the molar mass of these triblock copolymers used was very much lower than the ones we used. In this paper mechanical properties in the wet state and water uptake was not determined. In our case, we used triblock copolymers with much higher molar masses. This leads to materials with excellent processability, and very good mechanical properties in the hydrated state. We also illustrate their processability by extrusion-based 3D-printing using conventional commercially available equipment.

## 2. Materials and Methods

### 2.1. Materials

Polyethylene glycol (PEG) with number average molecular weights (Mn) of 6 kg/mol, 10 kg/mol and 14 kg/mol, 1,4-diazabicyclo[2.2.2]octane (DABCO) and hexamethylene diisocyanate (HDI) were purchased from Sigma Aldrich (St. Louis, MO, USA). PEG was dried by azeotropic distillation with toluene before use. Diphenyl phosphate (DPP) was obtained from Tokyo Chemical Industry (Oxford, UK). ɛ-caprolactone (CL) monomer was obtained from Acros Organics (Geel, Belgium) and was purified by drying over CaH_2_ (Merck, Darmstadt, Germany) and distillation under vacuum before use. Tetrahydrofuran (THF, from Merck, Germany) was dried over molecular sieves before use. Dichloromethane, methanol and diethyl ether were obtained from VWR Chemicals (Amsterdam, The Netherlands) and used as received. Deuterated chloroform (CDCl_3_) was purchased from Sigma Aldrich (USA).

### 2.2. Methods

#### 2.2.1. Synthesis of PCL-*b*-PEG-*b*-PCL and TPU-(PCL-*b*-PEG-*b*-PCL) Polymers

**Figure 1:**
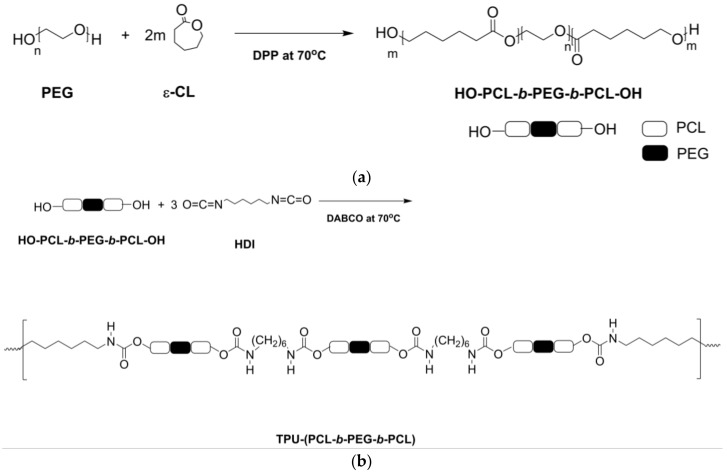
Synthesis of (**a**) hydroxyl-group terminated HO-PCL-*b*-PEG-*b*-PCL-OH triblock copolymers and (**b**) corresponding TPU-(PCL-*b*-PEG-*b*-PCL) thermoplastic polyurethane multi-block copolymers.

PCL-*b*-PEG-*b*-PCL triblock copolymers with targeted molecular weights of 20 kg/mol were prepared by ring opening polymerization (ROP) of ɛ-CL using PEG as an initiator and DPP as a catalyst as illustrated in Figure 1a. The molar ratio of DPP catalyst to PEG initiator was 1:1. The polymerizations were carried out under inert conditions (using argon gas) at 70 °C on a 50 g scale in 3-necked glass flasks equipped with mechanical stirrers. In a typical polymerization procedure, the dried PEG is introduced into the flask and heated under vacuum to 130 °C for 3 h. The temperature is then decreased to 70 °C, and freshly distilled ɛ-CL and DPP are added. While stirring, the reaction mixture was let to polymerize under at 70 °C for 96 h. The obtained polymer was dissolved in dichloromethane, precipitated in cold methanol and dried under vacuum.

A series of PCL-*b*-PEG-*b*-PCL triblock copolymers (PCL_7_-PEG_6_-PCL_7_, PCL_5_-PEG_10_-PCL_5_, PCL_3_-PEG_14_-PCL_3_) with different PCL block lengths (PCL_7_, PCL_5_, PCL_3_) was prepared in this manner using PEG with molecular weights of 6, 10 and 14 kg/mol (PEG_6_, PEG_10,_ PEG_14_).

Thermoplastic polyurethanes (TPUs, TPU-(PCL-*b*-PEG-*b*-PCL) were prepared by reacting the hydroxyl end-groups of the PCL-*b*-PEG-*b*-PCL triblock copolymers with hexamethylene diisocyanate (HDI) using 1,4-diazabicyclo[2.2.2]octane (DABCO) as a catalyst, see Figure 1b. The dried triblock copolymers were dissolved in anhydrous THF at a concentration of 70 g/mL at 70 °C in glass 3-necked flasks under argon, after which DABCO (2 wt.% relative to the polymer) was added.

Hexamethylene diisocyanate was then added slowly to the solution at a rate of 10 drops/10 min; the ratio of hydroxyl groups to isocyanate groups was 1:3. The reaction was continued for another 24 h. After slowly cooling to room temperature, the polymer solution precipitated in cold diethyl ether. After washing with water, the obtained polymer was dried under vacuum.

#### 2.2.2. Characterization of PCL-*b*-PEG-*b*-PCL and TPU-(PCL-*b*-PEG-*b*-PCL) Polymers

The chemical composition and the molar mass (Mn) of the triblock copolymers and the thermoplastic polyurethanes were determined by NMR-spectroscopy using a 400 MHz ^1^H-NMR apparatus (Bruker Ascend 400, Bruker, San Jose, CA, USA). ^13^C-NMR experiments at 100 MHz (Bruker Ascend 400) were conducted as well. The polymers were dissolved in deuterated chloroform, although it should be noted that in the case of the TPUs, a very small amount of minute fragments seemed to not have dissolved. These microgel particles could be the result of allophanate side reactions [21]. The chemical structure of the TPU block copolymers was additionally confirmed by ATR-FTIR using a Perkin Elmer Spectrum Two™ FTIR Spectrometer (Waltham, MA, USA). Spectra of polymer films were obtained using 32 scans at a resolution of 4 cm^−1^.

The inherent viscosity of the polymers was determined using a capillary Ubbelohde viscometer at 25 °C at a concentration of 0.1 g/dL in chloroform. Here too, very small amounts of minute swollen polymer fragments could be observed in the polymer solutions.

Differential Scanning Calorimetry (DSC) analyses were performed using a Pyris 1 DSC (Perkin Elmer, Waltham, MA, USA). Polymer samples (5–10 mg) were heated in stainless steel pans from −100 °C to 250 °C at a rate of 10 °C/min, rapidly cooled to −100 °C at 20 °C/min, after which a second scan was taken at a rate 10 °C/min. The glass transition (Tg) and the melting temperature (Tm) were determined using data from the second heating run.

#### 2.2.3. Physical and Mechanical Properties of PCL-*b*-PEG-*b*-PCL and TPU-(PCL-*b*-PEG-*b*-PCL) Polymers

Polymer films were fabricated by compression molding the polymers into sheets at 70 °C for 10 min using a hot press (THB 400, Fontijne BV, Barendrecht, The Netherlands), followed by cooling to room temperature. The thickness of the films was 200 to 300 µm. The water uptake of the TPU-(PCL-*b*-PEG-*b*-PCL) films (approximately 200 mg) was determined after equilibrium swelling in an excess of water (15 mL) at 37 °C using the following equation:

(1) $$\mathrm{water}\;\mathrm{uptake}=\left(\frac{m_{wet}-m_{dry}}{m_{dry}}\right)\times 100$$

where *m_dry_* and *m_wet_* are the mass of the dry film and that after it has swollen to equilibrium, respectively. The surface of the water-swollen samples was blotted prior to the measurement of their mass.

The tensile properties and the resistance to tearing of the TPU-(PCL-*b*-PEG-*b*-PCL) films in the dry and wet state were determined at room temperature using a universal tensile tester (Zwick ZO20, Ulm, Germany) equipped with a 500 N load cell. Tensile testing was done at room temperature in accordance to ASTM D882-91. Specimens measuring 100 × 5 mm^2^ were punched out from the films and tested at a crosshead speed of 50 mm/min. The E-modulus, the yield stress (σ_yield_), the elongation at yield (ɛ_yield_), the maximum tensile stress (σ_max_), the elongation at the break (ɛ_break_) and the toughness of the specimens (W in N/mm^2^, the surface area below the stress-strain curve), were determined from the stress-strain curves. The yield stress and -strain were determined from the intersection of the tangents to the stress-strain curves.

The average-(TPS_ave_) and maximal tear propagation strength (TPS_max_) of the polymers were determined according to ASTM D1938 at a crosshead speed of 250 mm/min using trouser tear specimens (75 × 25 mm^2^, with a 50 mm incision). The tearing energy (*G* expressed in kJ/m^2^) was calculated according to [22]:

(2) $$G=\frac{2F}{t}$$

where F is the average tearing force and t is the thickness of the specimen.

#### 2.2.4. Fabrication of Designed Structures by Fused Deposition Modelling (FDM)

Compression molded polymer films were cut into small pieces and extruded at 120 °C into filaments with a diameter of approximately 2.5 mm using a Noztek Pro filament extruder (Noztek, Shoreham, UK). The extruded filaments were collected on a rotating spindle and air-cooled to room temperature. The resulting filaments were then further processed by FDM into designed structures using an Ultimaker 2^+^ 3D Printer (Ultimaker, Geldermalsen, The Netherlands). Printing was done at 180 °C through a 0.4 mm nozzle at a printing speed of 1 mm/s, the building plate was at room temperature. The fabricated structures were imaged by scanning electron microscopy (HR-SEM, Zeiss 1550, Jena, Germany) after sputter-coating with gold.

## 3. Results

### 3.1. PCL-b-PEG-b-PCL Triblock Copolymers

Triblock copolymers were prepared by the ring opening polymerization of ɛ-CL with PEG as an initiator and DPP as a catalyst as described earlier. ^1^H-NMR analysis was used to determine monomer conversion, composition and molar mass of the triblock copolymers formed.

**Figure 2:**
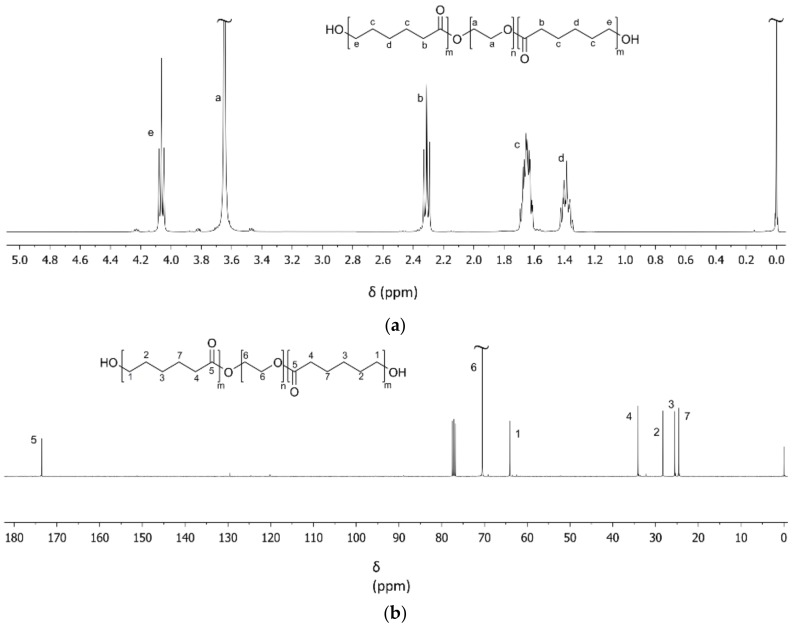
Characteristic (**a**) ^1^H-NMR and (**b**) ^13^C-NMR spectra of PCL_5_-PEG_10_-PCL_5_ triblock copolymers after purification by precipitation.

Figure 2a shows a characteristic ^1^H-NMR spectrum of a PCL_5_-PEG_10_-PCL_5_ triblock copolymer obtained after polymerization, precipitation and drying. The sharp singlet at 3.65 ppm (**a**) corresponds to the methylene protons of the PEG block. The triplet at 2.29 ppm corresponds to the –O–CO–C**H_2_**–CH_2_–CH_2_–CH_2_–CH_2_– (**b**) methylene protons of the PCL blocks, the multiplet from 1.59 to 1.68 ppm to the –O–CO–CH_2_–C**H_2_**–CH_2_–C**H_2_**–CH_2_– (**c**) methylene protons of the PCL blocks, the multiplet from 1.33 to 1.41 to the –O–CO–CH_2_–CH_2_–C**H_2_**–CH_2_–CH_2_– (**d**) methylene protons of the PCL blocks, and the triplet at 4.05 ppm to the –O–CO–CH_2_–CH_2_–CH_2_–CH_2_–C**H_2_**– (**e**) methylene protons of the PCL blocks. From the integral values of these peaks, the composition of the triblock copolymers can be calculated. Furthermore, knowing the molar mass of the PEG initiator as determined by ^1^H-NMR and assuming each PEG chain initiates the polymerization of CL, the molar mass of the PCL blocks and that of the triblock copolymer can be determined. An overview of the characteristics of the synthesized triblock copolymers is presented in Table 1. It can be seen that the molar mass and composition of the triblock copolymers was close to the targeted values.

The table also shows that the conversion of CL was very high. These values were determined from ^1^H-NMR spectra of the triblock copolymers before precipitation and drying (spectra not shown). The ε-CL conversion can be calculated from the integral values of the characteristic –O–CO–C**H_2_**–CH_2_–CH_2_–CH_2_–CH_2_– triplet of the ε-CL monomer at 2.47 ppm and the integral values of the characteristic –O–CO–C**H_2_**–CH_2_–CH_2_–CH_2_–CH_2_– triplet of the PCL blocks (**b**) at 2.31 ppm.

The ^13^C-NMR spectrum in Figure 2b illustrates the block structure of the synthesized triblock copolymers. In the spectrum, the peak at 70.69 ppm corresponds to the methylene carbon atoms of PEG (**6**). The peak at 64.28 ppm corresponds to the –O–CO–CH_2_–CH_2_–CH_2_–CH_2_–**C**H_2_– (**1**), the peak at 28.48 ppm to the –O–CO–CH_2_–CH_2_–CH_2_–**C**H_2_–CH_2_– (**2**), the peak at 25.66 ppm to the –O–CO–CH_2_–CH_2_–**C**H_2_–CH_2_–CH_2_– (**3**), the peak at 34.25 ppm to the –OC–O–**C**H_2_–CH_2_–CH_2_–CH_2_–CH_2_– (**4**) and the peak at 24.71 ppm to the –O–CO–CH_2_–**C**H_2_–CH_2_–CH_2_–CH_2_– (**7**) methylene carbons of the PCL blocks of the triblock copolymer. The peak at 173.64 characterizes the carbonyl carbon atom –O–**C**O–CH_2_–CH_2_–CH_2_–CH_2_–CH_2_– (**5**) of the PCL blocks of the triblock copolymer.

An overview of the thermal characteristics of the prepared triblock copolymers is given in Table 2. It can be seen that in the dry state, a Tg of approximately −60 °C can be observed. This temperature is close to the Tg of both PEG and PCL (respective values are approximately −55 °C and −60 °C). Distinct glass transition temperatures corresponding to the different amorphous phases of PEG and PCL could thus not be observed. In addition, single peak melting temperatures were seen, with Tm values close to 50 °C. The values of the melting enthalpies seem to decrease with decreasing PEG content. It was observed that upon cooling, the triblock copolymers crystallize at temperatures between 15 and 20 °C. The triblock copolymers with the lower PEG contents had the lower crystallization temperatures.

Films prepared by compression molding of these triblock copolymers were very fragile and fragmented; their mechanical properties were negligible. Furthermore, upon immersion in water they further fell apart into small pieces.

### 3.2. TPU-(PCL-b-PEG-b-PCL) Multi-Block Copolymers

TPUs based on the PCL-*b*-PEG-*b*-PCL triblock copolymers were successfully synthesized by step growth polymerization of the hydroxyl-terminated PCL-*b*-PEG-*b*-PCL triblock copolymers with HDI. These polymerizations led to multi-block polyurethanes with high molecular weights. The inherent viscosities of TPU-(PCL_3_-PEG_14_-PCL_3_), TPU-(PCL_5_-PEG_10_-PCL_5_) and TPU-(PCL_7_-PEG_6_-PCL_7_) were respectively 1.2, 1.2 and 1.4 dL/g.

**Figure 3:**
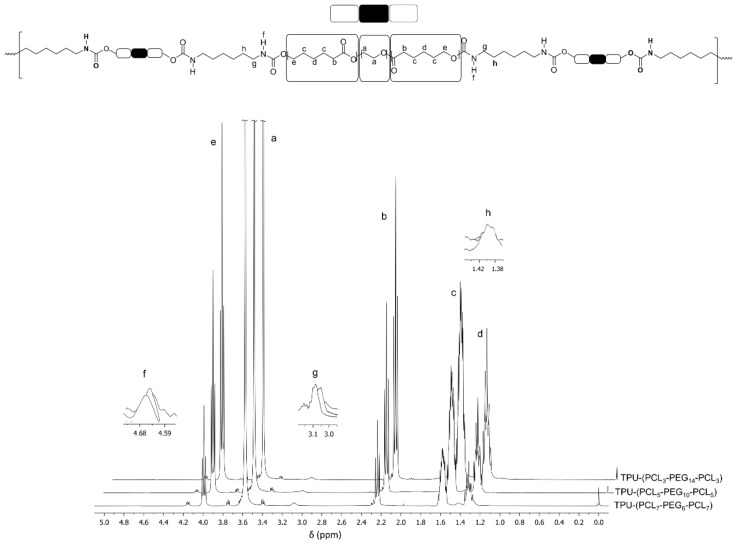
^1^H-NMR spectra of TPU multi-block copolymers based on different PCL-*b*-PEG-*b*-PCL triblock copolymers.

Figure 3 shows the characteristic ^1^H-NMR spectra of TPUs based on PCL-*b*-PEG-*b*-PCL triblock copolymer after purification. All individual signals that correspond to the PEG and PCL segments of the consistent triblock copolymer, as well as those of the formed urethane bonds, can be observed.

The sharp singlet at δ = 3.65 ppm is due to the methylene protons of the PEG component (a) of the triblock copolymer. The triplets at δ = 4.06, 2.31 and the multiplets at δ = 1.65 and 1.40 ppm relate respectively to the (e), (b), (c) and (d) methylene protons of the PCL block in the block copolymer. The urethane group, –OOCNH–, is clearly presented at δ = 4.68 (f) ppm.

**Figure 4:**
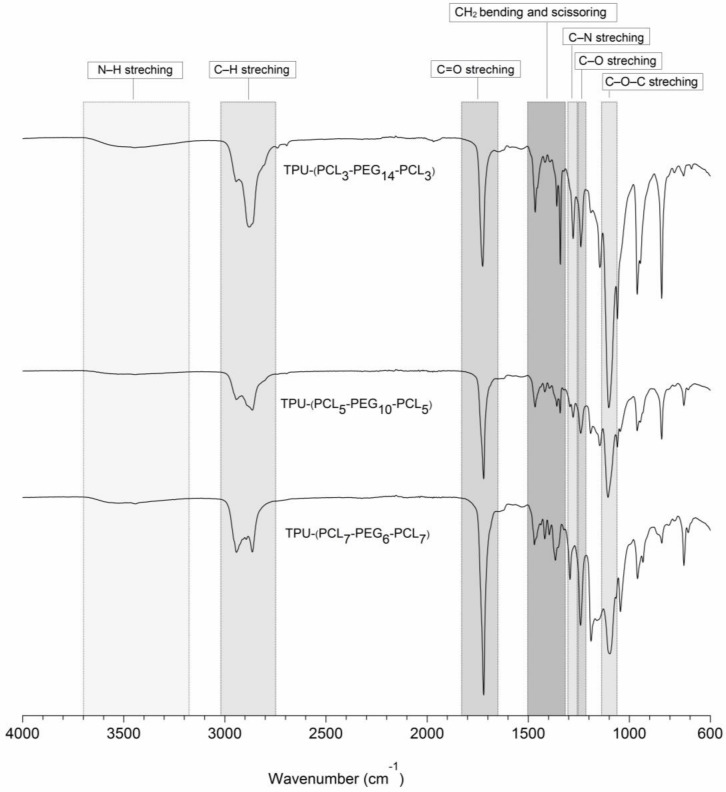
FTIR spectra of synthesized TPUs based on PCL-*b*-PEG-*b*-PCL triblock copolymers.

Additionally, FTIR analyses indicated the successful formation of urethane bonds upon reaction of the terminal hydroxyl groups of the triblock copolymers with hexamethylene diisocyanate. Figure 4 shows the FTIR spectra from 4000–600 cm^−1^ of the different synthesized and purified TPUs. The presence of urethane bonds is confirmed by the presence of a broad peak at 3700–3400 cm^−1^ that can be ascribed to N–H stretching vibrations. The absorption peak at 1270 cm^−1^ arises from contributions of the N–H in-plane bending and the C–N stretching vibrations. The absorption peak at 1244 cm^−1^ corresponds to the C–O stretching vibration mode of urethane bonds. The absence of a characteristic peak related to NCO isocyanate groups around 2270 cm^−1^ indicates that all NCO groups of HDI have reacted with the terminal OH groups of the precursor triblock copolymers.

Besides this, the absorption peaks at 2939 and 2864 cm^−1^ can be attributed to anti-symmetric and symmetric modes of the C–H stretching vibrations in the PEG and PCL segments. TPU multi-block copolymers based on PCL-*b*-PEG-*b*-PCL triblock copolymers containing higher PEG contents showed higher absorption peaks for the symmetric mode of the C–H stretching vibrations at 2864 cm^−1^. In addition, the C–O–C stretching vibration in the PEG part shows an absorption peak at 1100 cm^−1^. The C=O stretching vibration displays an absorption peak at 1723 cm^−1^; copolymers containing higher contents of PCL content show higher intensities of the C=O peaks. The absorption peaks at 1468, 1365 and 1348 cm^−1^ are related to CH_2_ bending and scissoring vibrations of the methylene groups in the PEG and PCL segments.

These FTIR results, together with the ^1^H-NMR and ^13^C-NMR data, confirm the successful formation of TPU-(PCL-*b*-PEG-*b*-PCL) multi-block copolymers.

**Figure 5:**
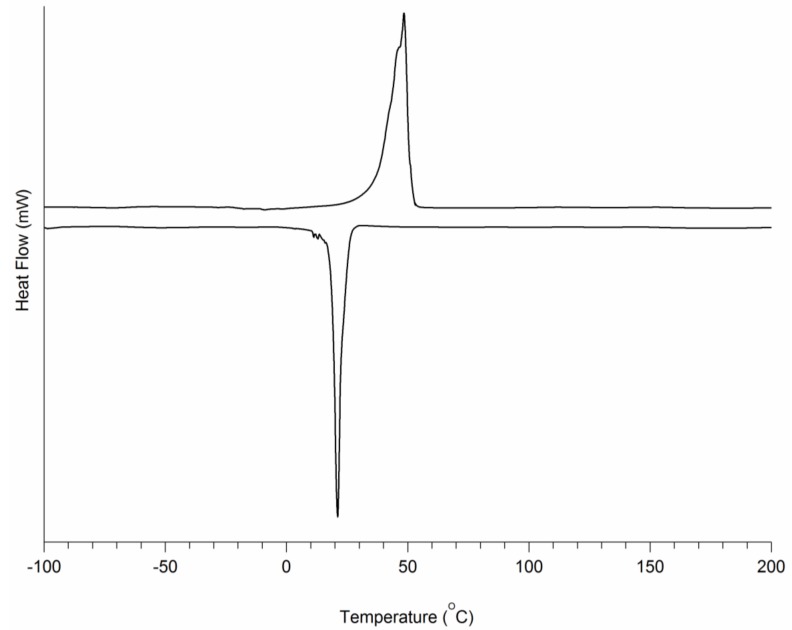
DSC thermogram of TPU-(PCL_5_-PEG_10_-PCL_5_) in the dry state. The sample was heated from −100 °C to 200 °C at 10/min, then cooled at a rate of 10/min.

An overview of the thermal characteristics of the obtained TPUs is listed in Table 3. A typical DSC graph is presented in Figure 5. It can be seen that in the dry state, a single Tg of approximately −60 °C can also be observed. In addition, single peak melting temperatures close to −50 °C were seen. The values of the melting enthalpies decrease with decreasing PEG content. It can be seen that the crystallization temperatures of the dry TPU-(PCL-*b*-PEG-*b*-PCL) multi-block copolymers is slightly above or close to room temperature. This implies that upon thermal processing and cooling to room temperature, crystallization will occur at maximum rates. Water uptake (equilibrium water uptake is reached in 3 days) is mainly due to the hydrophilic PEG component of the TPU-(PCL-*b*-PEG-*b*-PCL). The higher the PEG fraction in the TPU is, the higher the water uptake is. The melting temperatures and melting enthalpies of the TPUs in the wet state are mainly due to the PCL fraction and are significantly reduced when compared to the values in the dry state.

**Figure 6:**
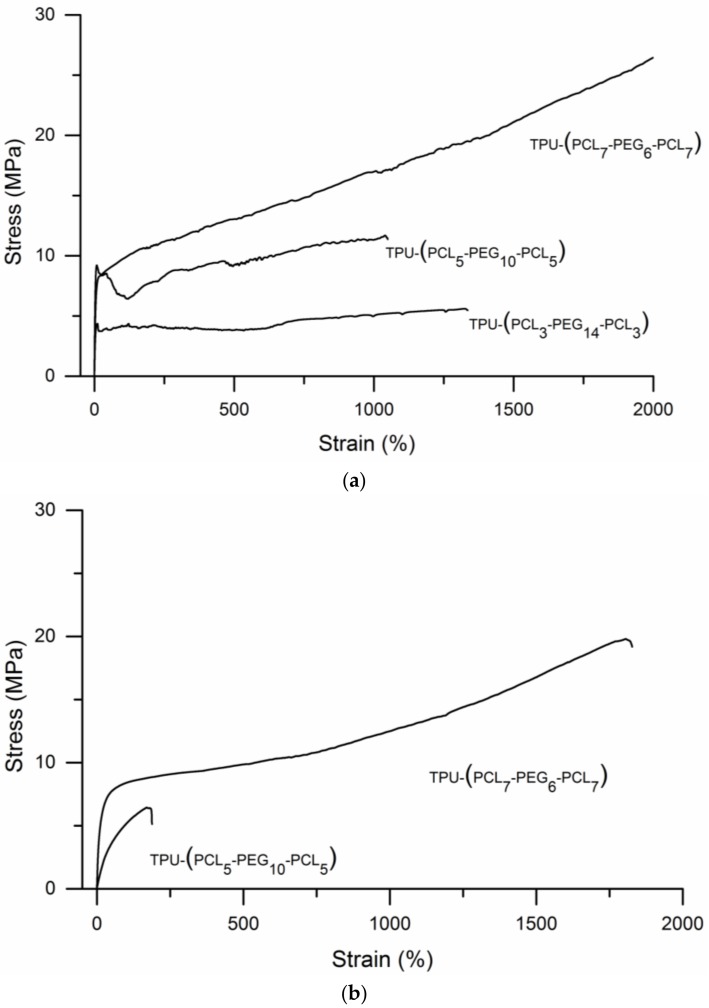
Stress-strain curves of the different TPU-(PCL-*b*-PEG-*b*-PCL) multi-block copolymers with different compositions: (**a**) Experiments conducted using dry specimens and (**b**) experiments conducted using wet specimens. The water uptake of TPU-(PCL_5_-PEG_10_-PCL_5_) was 534%, the water uptake of TPU-(PCL_7_-PEG_6_-PCL_7_) was 122%.

The tensile stress–strain behavior of the TPUs under dry and hydrated conditions are presented in Figure 6, quantitative data are given in Table 4. Figure 6a shows that under dry conditions all TPUs show plastic deformation and very high elongations at break. Values of the elasticity modulus, tensile yield stress and maximum tensile stress of the TPUs increase with PCL content. In addition, tensile toughness, the area under the stress-strain curves, increases significantly with PCL content (see Table 4).

Upon uptake of water, the elasticity modulus decreases. TPU-(PCL_5_-PEG_10_-PCL_5_) with a PEG content of 50% takes up 534% water. Values of its elasticity modulus, yield strength, maximum tensile strength, elongation at break and toughness significantly decrease when compared to the dry state. By comparison, TPU-(PCL_5_-PEG_10_-PCL_5_) takes up 122% water and only the value of the elasticity modulus decreases, while yield strength, maximum tensile strength, elongation at break and toughness change only to a limited extent. TPU-(PCL_3_-PEG_14_-PCL_3_) multi-block copolymers dissolve in water. Note that the swollen hydrogels remain stable for more than one month.

The toughness of the different TPU-(PCL-*b*-PEG-*b*-PCL) multi-block copolymers was also assessed in tearing experiments. Trouser tear propagation tests were performed in the dry- and in the wet-state, Table 5 gives an overview of the obtained results of the average tear propagation strength values, the maximum tear propagation strengths and the tearing energy. In the dry state TPUs TPU-(PCL_5_-PEG_10_-PCL_5_) and TPU-(PCL_7_-PEG_6_-PCL_7_) show comparable values of TPS_ave_, TPS_max_ and G. In the wet state, TPU-(PCL_5_-PEG_10_-PCL_5_) is significantly less tough than in the dry state. Most interestingly, the high toughness of TPU-(PCL_7_-PEG_6_-PCL_7_) is maintained in the hydrated state. It’s important to note that both in the dry and in the wet state these TPU hydrogels compare very favorably to other hydrogel materials [23,24]. The tear propagation strengths and tearing energy of TPU-(PCL_3_-PEG_14_-PCL_3_) could only be determined in the dry state, where its toughness was much lower than that of the other TPU-(PCL-*b*-PEG-*b*-PCL) multi-block copolymers.

### 3.3. 3D Printing of TPU-(PCL-b-PEG-b-PCL) Multi-Block Copolymers

**Figure 7:**
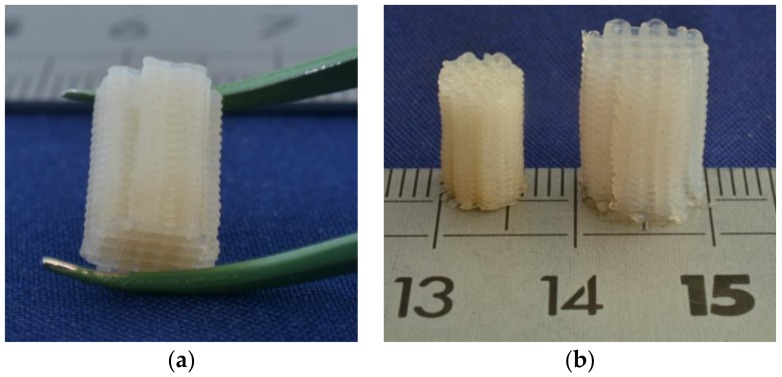
Designed structure prepared from TPU-(PCL_5_-PEG_10_-PCL_5_) multi-block copolymer by fused deposition modelling: (**a**) Structure in the dry state and (**b**) comparison of the structure in the dry state and in the hydrated state.

Designed tissue engineering scaffolding structures were prepared by fused deposition modelling (FDM) of the TPU-(PCL_5_-PEG_10_-PCL_5_) multi-block copolymer, as this thermoplastic hydrogel shows very high water uptake and excellent mechanical properties in the wet state. This TPU has a melting temperature of approximately 49 °C and a decomposition temperature of approximately 400 °C, as determined by thermogravimetric analysis (details not shown). To allow processing using a conventional commercially available FDM machine, filaments with a diameter of approximately 2.5 mm were first prepared by melt extrusion at 120 °C. The TPU filaments were then fed into the FDM apparatus to prepare the designed structures as described in the experimental part. The conditions were not thoroughly optimized, but an extrusion temperature of 180 °C, a nozzle diameter of 0.4 mm and a printing speed of 1 mm/s were found to be well suited. The building platform was kept at a temperature near room temperature, as this is close to the maximum crystallization temperature (see Table 2). Figure 7a shows one of the 3D printed structures. 

Figure 7b compares the prepared structure in the dry state and in the wet state after taking up large amounts of water. The structure swells homogeneously in all directions, and features of the designed structure such as its geometry, struts and pores are enlarged upon hydration. While the 3D-printed structure is relatively rigid in the dry state, it is quite flexible and elastic in the hydrated state. After compression, it fully returns to its original shape.

**Figure 8:**
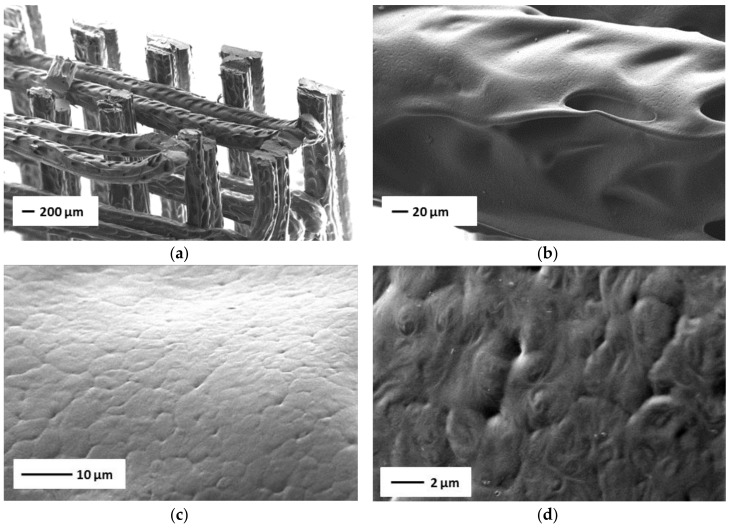
SEM images of 3D printed structures prepared from TPU-(PCL_5_-PEG_10_-PCL_5_) in the dry state at different magnifications. (**a**) overview of a printed structure, (**b**–**d**) images of the surface of the printed structure at higher magnifications.

TPU-(PCL-*b*-PEG-*b*-PCL) hydrogels are tough thermoplastic materials, consisting of hydrophilic PEG segments and hydrophobic PCL domains. Both components are crystallizable, but in the hydrated state it is the hydrophobic PCL domains that ensure the maintenance of the physical network. Figure 8 shows an overview of another 3D printed structure prepared from TPU-(PCL_5_-PEG_10_-PCL_5_) in the dry state. In Figure 8a, an image of the overall structure is shown, where the fibers/strands have a width of approximately 200 μm and the distance between the strands is close to 300 μm. Figure 8b is a higher magnification of one of the strands. The higher magnifications of the SEM images in Figure 8c,d shows that the surface of the printed strands is not smooth, and that spherulitic crystalline structures with diameters of 5–7 μm are present.

## 4. Conclusions

Thermoplastic polyurethanes based on PCL-*b*-PEG-*b*-PCL triblock copolymers can be prepared by a reaction of the triblock copolymers with hexamethylene diisocyanate. Depending on their PEG content, the synthesized TPU-(PCL-*b*-PEG-*b*-PCL) materials can take up large amounts of water of more than 500%. This allows them to be used as biodegradable thermoplastic hydrogels. Furthermore, these materials were shown to have excellent mechanical properties displaying high tensile strength, elongation at break, toughness and tear resistance in the dry as well as in the highly water-swollen state. This work has shown that these advanced thermoplastic materials can readily be processed into designed hydrogel structures by fused deposition modelling using conventional equipment, thus allowing them to be used in a variety of biomedical applications. Future work will address optimization of the printing process and the properties of built structures in more detail.

## Figures and Tables

**Table 1:** The composition and PEG and PCL block lengths of synthesized PCL-*b*-PEG-*b*-PCL triblock copolymers as determined by ^1^H-NMR after purification.

Target Structure	M_n_ of PEG Block ^a^	CL Conversion ^b^	M_n_ of PCL Blocks	PEG Content	M_n_ of Triblock Copolymer
	(kg/mol)	(%)	(kg/mol)	(mol%)	(kg/mol)
PCL_3_-PEG_14_-PCL_3_	13.9	99.2	½ × 5.9	70.2	19.8
PCL_5_-PEG_10_-PCL_5_	9.9	99.4	½ × 10.0	49.8	19.9
PCL_7_-PEG_6_-PCL_7_	5.9	99.8	½ × 14.1	29.5	20.0

^a^ M_n_ of PEG used as an initiator in the ring opening polymerization of CL. ^b^ CL conversion was determined before purification.

**Table 2:** Thermal characteristics of PCL-*b*-PEG-*b*-PCL triblock copolymers in the dry state.

	Tg (°C)	Tc (°C)	Tm (°C)	ΔH (J/g)
PCL_3_-PEG_14_-PCL_3_	−61	20	51	115
PCL_5_-PEG_10_-PCL_5_	−64	17	52	101
PCL_7_-PEG_6_-PCL_7_	−67	15	47	69

**Table 3:** Thermal characteristics of TPU-(PCL-*b*-PEG-*b*-PCL) in the dry and in the wet state.

		Water Uptake (wt.%)	Tg (°C)	Tc (°C)	Tm (°C)	ΔH (J/g)
DRY	TPU-(PCL_3_-PEG_14_-PCL_3_)	-	−58	26	48	100
	TPU-(PCL_5_-PEG_10_-PCL_5_)	-	−60	21	49	81
	TPU-(PCL_7_-PEG_6_-PCL_7_)	-	−60	23	51	37
WET	TPU-(PCL_3_-PEG_14_-PCL_3_) ^a^	soluble	-	-	-	-
	TPU-(PCL_5_-PEG_10_-PCL_5_)	534	−73	^b^	42	10
	TPU-(PCL_7_-PEG_6_-PCL_7_)	122	−69	^b^	49	19

^a^ Soluble in water. ^b^ Only crystallization of water can be discerned.

**Table 4:** Tensile properties of TPU-(PCL-*b*-PEG-*b*-PCL) multi-block copolymers in the dry and in the wet state. (*n* = 5, average ± SD).

	PEG/PCL	Water Uptake	E	σ_max_	ɛ_break_	σ_yield_	ɛ_yield_	W_tensile_
	(mol/mol)	(wt.%)	(MPa)	(MPa)	(%)	(MPa)	(%)	(N/mm^2^)
TPU-(PCL_3_-PEG_14_-PCL_3_)	70.2/29.8	dry	100 ± 15	6.1 ± 0.4	1080 ± 242	3.7 ± 0.5	5.1 ± 0.2	6244 ± 725
TPU-(PCL_5_-PEG_10_-PCL_5_)	49.8/50.2	dry	176 ± 16	8 ± 2	766 ± 282	7.5 ± 1	3.2 ± 0.6	5865 ± 3224
TPU-(PCL_7_-PEG_6_-PCL_7_)	29.5/70.5	dry	103 ± 16	15 ± 7	1566 ± 326	6.9 ± 0.6	6.1 ± 0.8	21841 ± 8667
TPU-(PCL_3_-PEG_14_-PCL_3_) ^a^	70.2/29.8	soluble	-	-	-	-	-	-
TPU-(PCL_5_-PEG_10_-PCL_5_)	49.8/50.2	534	7 ± 2	4 ± 1	147 ± 41	3.0 ± 0.6	3.0 ± 0.5	513 ± 267
TPU-(PCL_7_-PEG_6_-PCL_7_)	29.5/70.5	122	52 ± 10	17 ± 2	1553 ± 155	7.3 ± 0.7	13 ± 2	17976 ± 3011

^a^ Soluble in water.

**Table 5:** Tear propagation resistance of the different TPU-(PCL-*b*-PEG-*b*-PCL) polymers in the dry and in the wet state (*n* = 5, average ± SD).

	TPS_ave_ (N/mm)		TPS_max_ (N/mm)		G (kJ/m^2^)	
	Dry	Wet	Dry	Wet	Dry	Wet
TPU-(PCL_3_-PEG_14_-PCL_3_)	44 ± 12	^a^	71 ± 4	^a^	89 ± 12	^a^
TPU-(PCL_5_-PEG_10_-PCL_5_)	66 ± 14	8 ± 2	94 ± 3	13 ± 4	132 ± 21	16 ± 4
TPU-(PCL_7_-PEG_6_-PCL_7_)	64 ± 18	36 ± 20	93 ± 13	50 ± 24	127 ± 31	72 ± 41

^a^ Specimens take up large amounts of water and are very fragile.
